# Lignin Promotes Mycelial Growth and Accumulation of Polyphenols and Ergosterol in *Lentinula edodes*

**DOI:** 10.3390/jof9020237

**Published:** 2023-02-10

**Authors:** Feifei Wu, Heqin Wang, Qiufeng Chen, Xiao Pang, Hao Jing, Lijun Yin, Xiuqing Zhang

**Affiliations:** 1 College of Food Science and Nutritional Engineering, China Agricultural University, Beijing 100083, China; 2 Academy of National Food and Strategic Reserves Administration, Beijing 100037, China

**Keywords:** lignin, shiitake mushroom, biomass, phenolic compound

## Abstract

It has been demonstrated that lignin was efficiently degraded by *Lentinula edodes* (*L. edodes*). However, the process of lignin degradation and utilization by *L. edodes* has not been discussed in detail. Therefore, the effects of lignin on *L. edodes* mycelium growth, chemical compositions, and phenolic profiles were investigated herein. It has been revealed that 0.10% lignin acted as the most effective concentration to accelerate mycelia growth, which yielded the highest biomass of 5.32 ± 0.07 g/L. Furthermore, a 0.10% concentration of lignin promoted the accumulation of phenolic compounds, especially protocatechuic acid, with peak value of 48.5 ± 1.2 μg/g. In contrast, the higher concentration of lignin (0.20%) exerted an inhibitory effect on the growth of *L. edodes*. Overall, the application of lignin at the optimal concentration of 0.10% could not only enhance the mycelial growth but also accumulate the phenolic acids and raise the nutritional and medical values of *L. edodes*.

## 1. Introduction

Lignin is a high-branched polymer primarily composed of three phenyl propane monomers, namely coniferyl alcohol (G), sinapyl alcohol (S), and coumaryl alcohol (H) via both aryl-ether and alkyl linkages [1]. As the most abundant aromatic compound in nature, lignin has been widely recognized as a prospective bioresource for various chemicals as well as biofuels [2,3]. In recent years, the targeted conversion and high-value utilization of lignin by microbiological approaches have attracted widespread attention at home and abroad. Microorganisms such as fungi and bacteria (actinomycetes) are capable of degrading lignin to varying degrees, with white-rot basidiomycetous fungus being most efficient and being able to completely degrade lignin to water and carbon dioxide by its extracellular ligninolytic enzymes [4]. Previously, a wide array of ligninolytic enzymes secreted by white-rot fungi, including laccase, lignin peroxidase (LiP), manganese peroxidase (MnP), versatile peroxidases (VP), catalase, as well as other oxidative enzymes have been characterized [5,6]. The molecular mechanisms of lignin degradation by white-rot fungi have also been studied. In brief, the biodegradation of lignin started from the formation of alkyl propane monomers, followed by the breakdown and ring cleavage of heterogeneous aromatics. The degradation products are generally phenolic compounds, including protocatechuic acid, vanillin, *p*-coumaric acid, ferulic acid, and gallic acid. Moreover, it was reported that specificity biosynthesis and bioconversion of lignin had been achieved through fungi (*Saccharomyces cerevisiae*, *Phanerochaete chrysosporium*, *Cutaneotrichosporon* yeast) and bacteria (*Escherichia coli*, *Pseudomonas putida*, *Sphingobium* sp. SYK-6, *Rhodococcus opacus*) [7,8,9,10,11]. These investigations aroused our in-depth exploration of whether the exogenous addition of lignin could promote the accumulation of phenolic compounds in the microbial mycelium.

In recent years, the multi-pharmacological effects of *Lentinula edodes* (*L. edodes*), including antioxidative, immunoregulatory, and anticancer properties, have been well documented, which were associated with their bioactive components, such as polysaccharides, phenolic compounds, ergosterols, and terpenoids [12,13]. *L. edodes* is one of the most common edible mushrooms all over the world and has been widely cultivated and consumed particularly in the Asian countries, owing to its high nutritional value and unique flavor [14,15]. As the typical white-rot fungi, *L. edodes* commonly grows on sawdust or wood logs by degrading lignocellulose in nature, whose three major components are cellulose, hemicellulose, and lignin. Enzymes involved in lignocellulose degradation as well as their encoding gene clusters have been identified in *L. edodes* previously [16,17,18].

Currently, lignocellulose derived from different sources is taken as the alternative to sawdust and utilized in *L. edodes* cultivation for the purpose of forest resource protection and sustainable development [19,20]. The proportion of cellulose, hemicellulose, and lignin varied with the sources and types of lignocellulose [21]. *L. edodes* presented diverse growth characteristics on different lignocellulose culture substrates. This was attributed to the individual metabolic characteristic of *L. edodes* on the three components of lignocellulose. It revealed that *L. edodes* prefers hemicellulose over cellulose as the carbon source [16]. Moreover, our previous study has proved that hemicellulose stimulated mycelia biomass and polysaccharides biosynthesis [22]. Meanwhile, it was found that there was a synergistic relationship between lignin and hemicellulose in lignocellulose degradation in *L. edodes* [23].

To date, there are many investigations focusing on the biodegradation of lignin as well as the interrelation between lignin, microorganism, and enzymes. Nevertheless, as the precursor of phenolic acids, whether the addition of lignin will affect the metabolism, transformation, and enrichment of phenolic acids in *L. edodes* mycelia remains to be further explored. Moreover, the impact of lignin on mycelium growth is still unclear during liquid fermentation. To this end, our report aimed to reveal the impacts of lignin on the fugal growth, chemical compositions, as well as accumulation of phenolic compounds in *L. edodes* during liquid fermentation.

## 2. Materials and Methods

### 2.1. Strain and Materials

The *L. edodes* strain Qiuzai No.7 (Q-7) used in the present study was obtained from Hubei Yuguo Guye Co., Ltd. (Suizhou, China) and was preserved on potato dextrose agar (PDA) medium at 4 °C. Sodium lignosulfonate and xylo-oligosaccharide were purchased from Aladdin Biotechnology Co., Ltd. (Shanghai, China) and Shandong Longlive Bio-Technology Co., Ltd. (Dezhou, China), respectively [22].

Ergosterol as well as phenolic compound standards (gallic acid, protocatechuic acid, caffeic acid, syringic acid, vanillin, p-coumaric acid, and ferulic acid) were purchased from Sigma-Aldrich (St. Louis, MO, USA). Acetonitrile and methanol with a purity of 99.9% as the mobile phase were of high-performance liquid chromatography (HPLC) grade (Merck, Germany). All other chemicals and solvents were of analytical grade and purchased from common suppliers.

### 2.2. Culture Condition

The basic liquid medium for *L. edodes* growth was a modified potato dextrose broth (PDB) medium containing 2% (*w*/*v*) hemicellulose instead of glucose [22]. Lignin was added to the medium at the beginning of cultivation to provide a final concentration ranging from 0.05%, 0.10%, and 0.20% (*w*/*v*), respectively, while the media without lignin served as a control (CK). Prior to autoclaving, the culture media were acidified to pH 6.0 ± 0.1 with 1 M HCl. Experimental mycelia were collected after cultivation for 10 days in a shaking incubator (160 rpm) at 28 °C and 75% relative humidity (RH). RH in the laboratory was maintained at 50–70% constantly. The supernatants were analyzed for enzyme activity. All experiments were conducted in triplicates.

### 2.3. Determinations of Mycelium Biomass and Compositional Analysis

After 10 days fermentation, experimental mycelia were harvested by centrifugation (5000× *g*, 20 min) and then washed by deionized water sufficiently, followed by lyophilization. Mycelia yield was measured as follows:

y = M_1_/V_1_ (1)

in which y is the mycelia or biomass yield (g/L), M_1_ is the dry weight of mycelia (g), and V_1_ is the volume of medium (L).

The chemical compositions of cultured mycelia, including crude protein, total polysaccharide, ergosterol, and ash, were determined according to the AOAC methods (AOAC, 1995). Briefly, ash content was measured by carbonization and incineration in a muffle furnace at 550 °C until constant weight. Crude protein content (N × 4.38) was determined according to the micro-Kjeldahl method. Total water-soluble polysaccharides (WSP) were extracted with boiling water and then precipitated with anhydrous ethanol (1:4, *v*/*v*) at 4 °C overnight. After centrifugation at 5000× *g* for 30 min, precipitate was washed three times with five volumes of ethanol, followed by deproteinization with Sevag reagent (1-butanol/chloroform, *v*/*v* = 1:4) and lyophilization to obtain the crude polysaccharide. The yields of crude WSP extracts were recorded subsequently. The content of ergosterol was measured according to the method by Niemenmaa, Galkin, and Hatakka (2008), with slight modifications [24]. In short, mycelia were saponified by 10% KOH in methanol at 80 °C for 1 h. The supernatant was mixed with hexane and distilled water and then centrifuged at 6000× *g* for 15 min. The preceding step was repeated twice, whereafter the n-hexane phase was collected and evaporated under vacuum at 50 °C. Extracted ergosterol was redissolved with 500 μL of methanol (HPLC grade) and filtered through a 0.22 µm membrane. Extracted ergosterol was redissolved with 500 μL of methanol and filtered through a 0.22 µm membrane. Agilent 1260 Infinity HPLC system coupled with a diode array detector (DAD) and a C_18_ column (4.6 × 250 mm, 5 µm; Agilent, Santa Clara, CA, USA) was employed. A total of 20 μL of sample solution was injected and eluted with methanol at a constant flow rate of 1.0 mL/min at 30 °C.

### 2.4. Phenolic Compounds Extraction and Determination

The total phenolic was extracted by 80% methanol and measured according to the Folin–Ciocalteu colorimetric method with gallic acid as a standard [25]. The results were expressed as mg of gallic acid equivalents (GAE) per g of dried mycelia. For the investigation of the phenolic compounds profile, phenolic compound extracts were prepared as described by Gbylik-Sikorska et al. (2019) with slight modifications [26]. A total of 1 g lyophilized mycelia were dispersed in 10 mL of acetonitrile and 2 mL of hydrochloric acid. The mixtures were extracted for 30 min and immersed in a water bath with ultrasonic at 30 °C for 2 h. After centrifugation, the supernatant was collected and evaporated to dryness. Residues were redissolved in methanol and filtered through a 0.22 μm PVDF membrane for subsequent analysis.

The phenolic compound analysis was conducted on an Agilent 1260 Infinity HPLC system equipped with a C_18_ column (4.6 × 250 mm, 5 µm; Agilent, Santa Clara, CA, USA). A diode array detector (DAD) was carried out, using 250 nm, 280 nm, and 320 nm as the preferred wavelengths. A total of 20 μL of the sample was injected and eluted with mobile solvents consisting of 0.1% formic acid in water (A) and acetonitrile (B) at 30 °C. For efficient separation, a gradient elution procedure at a flow rate of 1 mL/min was used as follows:

(2) $$5\%B\;\frac{\to }{10\;\min}15\%B\;\frac{\to }{10\;\min}\;25\%B\;\frac{\to }{15\;\min}\;35\%B\;\frac{\to }{5\;\min}\;80\%B\;\frac{\to }{5\;\min}\;5\%B$$

### 2.5. Enzyme Assays

Mycelia (1.00 g) were homogenized with phosphate buffer solution (PBS) buffer (4 mL, 0.1 M, pH 7.4) in an ice bath. The crude enzyme solution was prepared by collecting the supernatant after centrifugation for 30 min at 10,000× *g* and 4 °C.

The CAT activity was determined according to the manufacturer’s instruction using a commercial detection kit (Nanjing Jiancheng Bioengineering Institute, China) and expressed as U/mg protein. Total protein content in crude extracts was measured by the Bradford method with bovine serum albumin (BSA) as a standard.

The laccase (Lcc, EC 1.10.3.2) activity was spectrophotometrically determined according to the oxidation rate of 2,2-azino-bis-[3-ethyltiazoline-6-sulfonate] (ABTS) in 20 mM Na-acetate buffer (pH 4.5) in the absorbance at 420 nm. One unit of Lcc activity was defined as the amount of enzyme able to oxidize 1 μmol of ABTS per minute at 20 °C as described by Edae and Alemu (2017) [27].

The Mn-peroxidases (MnP, EC 1.11.1.13) activity was assayed with phenol red as the substrate, and absorbance was recorded at 610 nm as described by Lechner and Papinutti (2006) [28]. Briefly, the reaction solution was mixed with manganese sulfate (0.1 mM), phenol red (0.1 mM), and succinate buffer (50 mM, pH 4.5). The reaction was initiated with the addition of H_2_O_2_ (0.1 mM at final concentration). One unit of MnP activity was defined as the amount of enzyme catalyzing 1 μmol of phenol red in one min.

### 2.6. Statistical Analysis

All experiments were conducted in triplicate, and values were expressed as mean ± standard deviation (SD). Statistical analysis was carried out by one-way ANOVA accompanied by Duncan’s test using SPSS (version 20.0). The significance was set at *p* < 0.05 and expressed by different letters above the error bars.

## 3. Results and Discussions

### 3.1. Effect of Lignin on Mycelia Growth

**Figure 1:**
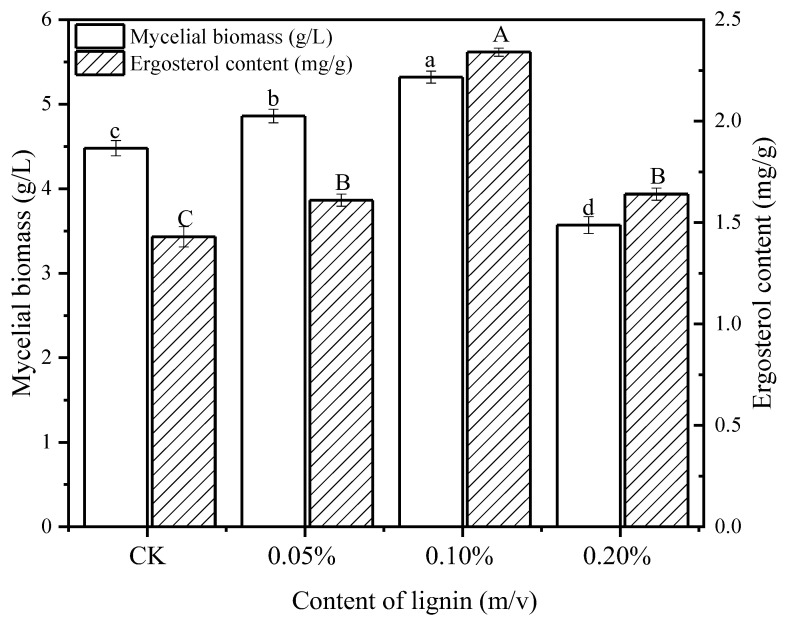
Effect of lignin on mycelial biomass and ergosterol content. Experiments were performed in triplicate, and results are presented as mean ± SD. Different letters (a–d, A–C) represented the significant differences in mycelial biomass and ergosterol content among samples (*p* < 0.05). Statistical analysis was carried out by one-way ANOVA accompanied by Duncan’s test using SPSS (version 20.0).

The effect of lignin on the mycelial growth of *L. edodes* was evaluated according to the harvested mycelial biomass, as shown in Figure 1. The yield of mycelial biomass refers to the dry weight (g) of mycelia produced per liter of culture medium. It suggested that the addition of lignin has significantly increased the mycelial biomass during 10 d cultivation in a concentration-dependent manner. The highest mycelial biomass (5.32 ± 0.07 g/L) was obtained at the concentration of 0.10%, with an increase of 20% compared with the control group (4.48 ± 0.09 g/L). However, supplementation with lignin at the concentration of 0.20% resulted in a significant decrease in mycelial biomass to 3.57 ± 0.09 g/L, indicating that a higher concentration of lignin might play an inhibitory role in *L. edodes* growth.

Corresponding with the mycelial biomass increase, the ergosterol content was enhanced due to the addition of lignin. The maximum value achieved was 2.34 ± 0.02 mg/g at the concentration of 0.1% lignin, which was 1.64 times that of the control group (1.43 ± 0.05 mg/g). Similarly, there was a downward trend of ergosterol content at the concentration of 0.2% lignin in comparison with that of 0.10% lignin.

Mycelial biomass is largely affected by culture conditions, including carbon and nitrogen sources, pH, temperature, and additives [29,30]. The addition of lignin was observed to promote fungal growth, in line with previous reports [31,32,33]. On the one side, it is likely that lignin accelerated the consumption rate of carbon source and energy metabolism in *L. edodes* in response to the elevated abundance and activity of hemicellulases [23,34]. Apart from this, vanillin, the intermediate product during lignin degradation, acted as an activator and stimulated the growth and proliferation of fungal cells [35]. Furthermore, considering that the biodegradation process was a cascade of oxidative reactions, various reactive oxygen species (ROS) would be generated and accumulated, leaving the *L. edodes* cells in an oxidative stress status. Thereby, an appropriate oxidative stress state (when the concentration of lignin is less than 0.20%) was inferred to promote *L. edodes* growth, which is consistent with a previous study [36]. Whereas, as reported by IIvashechkin et al. (2014), a culture medium containing lignin stimulated the growth of *L. tigrinus* simultaneously with a variation in the composition of phospholipids, suggesting that phosphatidic acid acted as a second messenger during the utilization of lignin by *L. tigrinus*. Therefore, the regulatory mechanism of lignin on the mycelium biomass of *L. edodes* remains to be further explored. In addition, it was deemed that declined mycelial biomass observed at the concentration of 0.20% resulted from the excess lignin-derived aromatic compounds present in the liquid medium, whose inhibitory effect on fungal growth and biochemical activities was discovered previously [37].

Ergosterol is the primary sterol of the fungal cell membrane and exists as a specific secondary metabolite in fungi proliferation [38]. As an indirect indicator for fungal growth, there was a positive correlation between ergosterol content and fungal biomass (Figure 1) [24]. Similarly, Vikman et al. (2002) discovered that bleached kraft paper that contained 0.2% lignin had an increased concentration of ergosterol in a compost inoculum [32]. As an aerobic process, biosynthesis of ergosterol is closely related to culture conditions and fungal growth status, with the level of dissolved oxygen as a vital parameter [39,40,41]. Noticeably, a higher concentration of oxygen was supplied in the liquid medium owing to the lignin degradation [36].

### 3.2. Effect of Lignin on Chemical Compositions of L. edodes Mycelium

The chemical composition of *L. edodes* mycelia cultured with lignin was determined, including total water-soluble polysaccharides (WSP), crude protein, and ash (Table 1). It indicated that these chemical compositions were greatly influenced by the presence of lignin in a concentration-dependent manner. Lignin-cultured *L. edodes* possessed the highest content of WSP (6.44 ± 0.33 g/100 g) at the concentration of 0.10%, reaching an increase of 2-fold in comparison to control (2.11 ± 0.14 g/100 g). This result was in line with the previous study [42]. Meanwhile, yields of WSP cultured by the supplementary lignin were higher than others reported by previous studies [14,43]. It is concluded that the culture medium consisting of 0.10% lignin was more favorable for the biosynthesis of polysaccharides in *L. edodes*.

Polysaccharides extracted by water have been proven to exhibit various bio-activities, such as antioxidative and immunoregulatory characteristics [22,44]. Regarding the synthesis of polysaccharides, it is closely correlated with the carbon sources and is enhanced with the increase in carbon source concentration [29]. For further utilization within the cell, hemicellulose was required to be degraded by means of glycosidic bond hydrolysis, which was accelerated by lytic polysaccharide monooxygenases (LPMOs) [45]. It is assumed that more electrons were provided to the LPMO active site owing to the low molecular lignin-derived products, which in turn boosted the hydrolysis of hemicellulose and facilitated the biosynthesis of polysaccharides.

It was also worth noting that crude protein contents in the mycelia of lignin-grown *L. edodes* were higher than that of the control. Meanwhile, the highest crude protein content (29.24% ± 0.09%) was detected when *L. edodes* was cultured with 0.10% lignin, with an enhancement of 13.2% versus that of the control group. The protein content was higher than that reported by Carneiro et al. (2013), which was 12.76% ± 0.24% for the marketing *L. edodes* [46]. Previous proteomic analysis expounded that in the presence of lignin, large amounts of enzymes involved in lignin degradation were secreted, such as manganese peroxidases, laccase, and lignin peroxidases [23]. Ash content gradually accumulated with the increasing lignin concentration, ranging from 3.78% to 6.14%. The variation in ash content might be ascribed to the higher mineral assimilation capacity stimulated by lignin in lignin-cultured mycelia. Uptake of the mineral from a modified PDB culture medium for *L. edodes* is a bioprocess containing the transport, exchange, complexation, and adsorption of ions [47].

### 3.3. Effect of Lignin on Phenolic Compounds Metabolism in Mycelia of L. edodes

**Figure 2:**
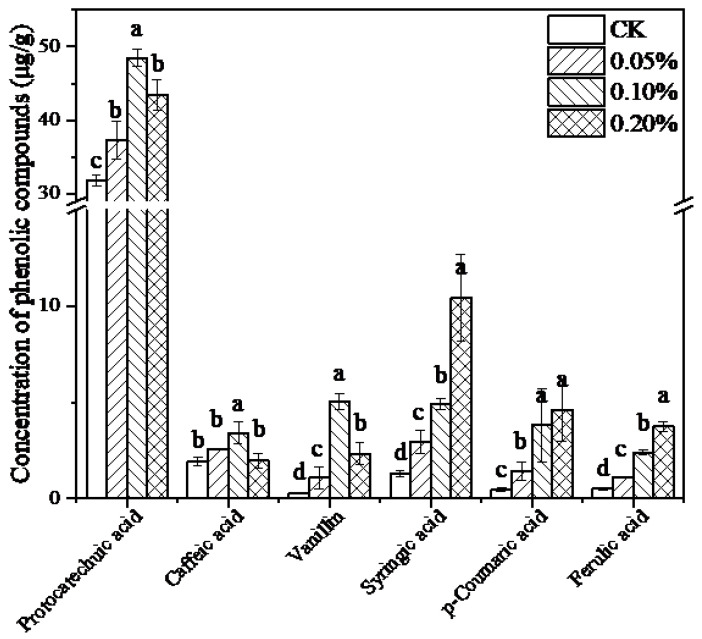
Contents of phenolic compounds from *L. edodes* mycelium cultured in lignin-containing medium. Results are presented as mean ± SD, n = 3. Different letters represented the significant differences among sample groups (*p* < 0.05). Statistical analysis was carried out by one-way ANOVA accompanied by Duncan’s test using SPSS (version 20.0).

Considering the complex structure composed of phenylpropanoid units linked by ether bonds and carbon–carbon bonds, depolymerization of lignin potentially released a variety of phenolic intermediates. Phenolic compounds in *L. edodes* mycelia with known pharmacological activities were detected by HPLC [48]. It was suggested that the addition of lignin had a significant influence on the phenolics in the *L. edodes* mycelium. Regarding the total phenolic content (Table 1), a gradual increase with increasing lignin concentration was observed, and the total phenolic content had a 2.9-fold increase as compared to that of the control. A total of six phenolic compounds were detected, including protocatechuic acid, caffeic acid, syringic acid, vanillin, *p*-coumaric acid, and ferulic acid, with contents varying ranged from 0.27 ± 0.04 mg/g to 48.49 ± 1.2 mg/g (Figure 2). It demonstrated that the addition of lignin led to a significant difference in phenolic compounds in different treatment groups (*p* < 0.05). A total of six phenolic compounds could be divided into two categories. The contents of protocatechuic acid, caffeic acid, and vanillin were increased at the concentration of 0.10%. It is worth noting that protocatechuic acid is the most abundant phenolic acid in the present study, whose level was an order of magnitude higher than others. However, the other three phenolic acids presented a different trend in which *p*-coumaric acid, ferulic acid, and syringic acid contents are correlated with lignin concentration.

**Figure 3:**
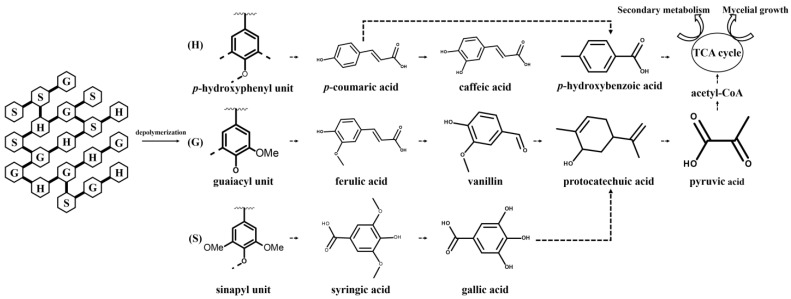
Schematic diagram of lignin degradation pathway in *L. edodes*. The dotted line represents an indirect reaction process. H, G, and S in the figure refer to three phenyl propane monomers of lignin, which named coumaryl alcohol (H), coniferyl alcohol (G), and sinapyl alcohol (S), respectively [49,50].

Contents of total phenolics in mycelium varied with the amount of lignin added, which resulted from the depolymerization of lignin and generation of monomer phenols in *L. edodes*. It was expected that different trends among the phenolic compounds resulted from the degradation pathway of lignin in *L. edodes*. Based on the obtained results, the putative pathway for the degradation process of lignin and transformation into phenols in *L. edodes* was outlined, referring to previous literature with slight modifications (Figure 3) [49,50]. Generally, the degradation process often occurred extracellularly, and low-molecular-weight monomers in the lignin polymer denoted as *p*-hydroxyphenyl (H), guaiacyl (G), and syringyl (S) were released, crossed the cell membrane and entered the cell [49]. The corresponding phenolic acids, referred to as *p*-coumaric acid, ferulic acid, and syringic acid, were investigated in the present study. As depicted in Figure 2, vanillin and *p*-coumaric acid are pivotal intermediates during the degradation process, while vanillin is formed from the conversion of ferulic acid and *p*-coumaric acid. The vanillin content in *L. edodes* mycelia without lignin was quite low. However, there was a 17.6-time increase after the addition of lignin. Vanillin served as an activator to accelerate the extracellular production of laccase and peroxidase. Moreover, the accumulation of vanillin contributed to the further conversion to downstream protocatechuic acid. Notably, protocatechuic acid is a conserved intermediate product of different monomers in most situations, acting as a “biological funnel” to limit the rate and efficiency of the lignin utilization process [51,52]. Ring cleavage of protocatechuic acid by dioxygenase enzymes occurred, yielding a large quantity of acetyl-CoA and pyruvate for entry into the TCA cycle ultimately. Based on this, it was convinced that the presence of lignin at a concentration of 0.1% raised the central metabolic level of *L. edodes*, offering more substrates and energies for fungal growth as well as secondary metabolites production. Moreover, the improved content of caffeic acid stemmed from the supplementary lignin. Caffeic acid has been identified to be widely distributed in several edible mushrooms [53]. Structurally, caffeic acid belongs to the hydroxycinnamic acid family, whose biosynthesis commonly takes *p*-coumaric acid as a precursor and employs *p*-coumarate-3-hydroxylase (C3H) to transfer a hydroxyl group onto the 3-position of the *p*-coumaric acid [54]. Hence, biotransformation among diverse phenols occurred in lignin-cultured *L. edodes*. Based on the experimental data, the addition of lignin improved the production of high-value phenolic compounds, especially lignin-derived phenols in *L. edodes*.

### 3.4. Effect of Lignin on Ligninolytic Enzymes in L. edodes Mycelia

**Figure 4:**
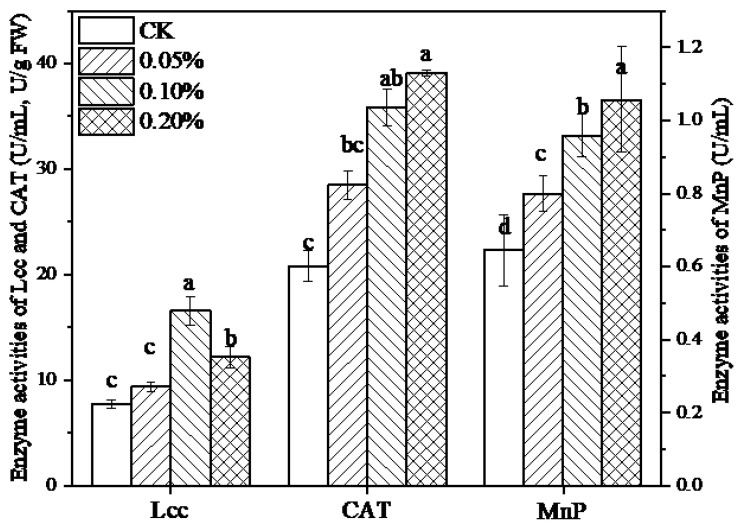
Enzyme activities of Lcc, CAT, and MnP in the absence or presence of lignin. Results are presented as means ± SD of three replicates. Different letters represented the significant differences among sample groups (*p* < 0.05). Statistical analysis was carried out by one-way ANOVA accompanied by Duncan’s test using SPSS (version 20.0).

Prior to further utilization, lignin was depolymerized via the potent extracellular ligninolytic enzymes in *L. edodes*. Activities of related enzymes, including Lcc, MnP as well as CAT, were evaluated in the presence of lignin (Figure 4). It turned out that 10 days of fermentation with lignin promoted the activities of three enzymes significantly (*p* < 0.05). Among them, the highest levels of laccase were observed at a concentration of 0.1% lignin, which was 2.1-fold higher compared to the levels recorded in the absence of lignin. In addition, lignin caused up to 1.65 and 1.88-fold increases in MnP and CAT activity, respectively, which reached maximum levels at 0.2% lignin. Meanwhile, a positive correlation was observed between MnP and CAT.

Several types of extracellular oxidative enzymes involved in the degradation of lignin in white-rot fungi have been elucidated thoroughly, including laccase and MnP, the most important ones in lignin depolymerization. Their activities and contents are closely related to the growth status of fungi in response to various culture conditions [55,56]. It has been reported that aromatic compounds, including ferulic acid and vanillin, which were blended in the present medium, stimulated both the Lcc and MnP secretions. In contrast, different strains performed diversely in response to different kinds of aromatic compounds related to their own specific signaling pathways of lignin and phenolic compounds in microorganisms [55]. Additionally, the low activity of MnP was confirmed in *L. edodes* previously, which might be accounted for by the lack of necessary substrate Mn^2+^ in the medium [57]. What is more, the production of enzymes in fungi is a dynamic course; thereby, cultivation time plays a vital role in MnP activities. Owing to the generation of large numbers of reactive oxygen species (ROS) during lignin degradation, the increase in the CAT activities mediated by lignin enabled to eliminate of free radicals in the fugal cell and to ease the oxidative stress, thereby ensuring the activation of fungal growth and secondary metabolite synthesis under high-level metabolic conditions.

## 4. Conclusions

In this work, lignin at a concentration of 0.1% (*w*/*v*) was found to promote fungal growth in liquid fermentation, accompanied by an increase in ergosterol, a biomarker of fungal biomass. At the same time, biosynthesis of proteins and WSP with multiple physiological activities were induced, which was possibly attributed to the presence of ROS or lignin derivatives generated during the lignin degradation process. With respect to the profile of phenolic compounds, changes of three monomers of syringic acid, *p*-coumaric acid, and ferulic acid were lignin-concentration-dependent, while the productions of protocatechuic acid, caffeic acid, and vanillin were the highest in 0.1% lignin-induced growth of *L. edodes*. Moreover, the activities of lignin-degrading enzymes such as Lcc, MnP, and CAT were increased. Additionally, Lcc and CAT played the primary roles in the degradation of lignin in *L. edodes* compared to MnP.

Overall, it was recommended that lignin induction was a promising approach for mycelia growth as well as phenolic acid accumulation in *L. edodes* liquid fermentation. Our present exploration provides insights into the exploitation and application of lignin as well as a foundation for the value-added cultivation of *L. edodes*.

## Figures and Tables

**Table 1:** Chemical composition of *L. edodes* cultured in the presence of lignin.

Lignin	WSP Extracts (g/100 g)	Crude Protein (*w*/*w* %)	Ash (*w*/*w* %)	Total Phenolics (mg GAE/g DW)
0	2.11 ± 0.14 ^d^	25.84 ± 0.35 ^c^	3.78 ± 0.07 ^d^	1.45 ± 0.03 ^d^
0.05%	4.14 ± 0.35 ^b^	27.63 ± 0.26 ^b^	4.89 ± 0.09 ^c^	2.28 ± 0.05 ^c^
0.10%	6.44 ± 0.33 ^a^	29.24 ± 0.09 ^a^	5.21 ± 0.11 ^b^	3.87 ± 0.14 ^b^
0.20%	2.94 ± 0.28 ^c^	26.17 ± 0.18 ^c^	6.14 ± 0.17 ^a^	5.62 ± 0.21 ^a^

In each column, different lowercase superscript letters represent significant differences between different treatments (*p* < 0.05). Statistical analysis was carried out by one-way ANOVA accompanied by Duncan’s test using SPSS (version 20.0). Results are presented as means ± SD of three replicated independent determinations.

## Data Availability

All data are available in the article.
